# Microtubule Actin Crosslinking Factor 1 Regulates the Balbiani Body and Animal-Vegetal Polarity of the Zebrafish Oocyte

**DOI:** 10.1371/journal.pgen.1001073

**Published:** 2010-08-19

**Authors:** Tripti Gupta, Florence L. Marlow, Deborah Ferriola, Katarzyna Mackiewicz, Johannes Dapprich, Dimitri Monos, Mary C. Mullins

**Affiliations:** 1 Department of Cell and Developmental Biology, University of Pennsylvania School of Medicine, Philadelphia, Pennsylvania, United States of America; 2 Generation Biotech, Lawrenceville, New Jersey, United States of America; 3 Department of Pediatrics, University of Pennsylvania School of Medicine, Philadelphia, Pennsylvania, United States of America; 4 Department of Pathology and Laboratory Medicine, The Children's Hospital of Philadelphia, Philadelphia, Pennsylvania, United States of America; Stanford University School of Medicine, United States of America

## Abstract

Although of fundamental importance in developmental biology, the genetic basis for the symmetry breaking events that polarize the vertebrate oocyte and egg are largely unknown. In vertebrates, the first morphological asymmetry in the oocyte is the Balbiani body, a highly conserved, transient structure found in vertebrates and invertebrates including *Drosophila*, *Xenopus*, human, and mouse. We report the identification of the zebrafish *magellan (mgn)* mutant, which exhibits a novel enlarged Balbiani body phenotype and a disruption of oocyte polarity. To determine the molecular identity of the *mgn* gene, we positionally cloned the gene, employing a novel DNA capture method to target region-specific genomic DNA of 600 kb for massively parallel sequencing. Using this technique, we were able to enrich for the genomic region linked to our mutation within one week and then identify the mutation in *mgn* using massively parallel sequencing. This is one of the first successful uses of genomic DNA enrichment combined with massively parallel sequencing to determine the molecular identity of a gene associated with a mutant phenotype. We anticipate that the combination of these technologies will have wide applicability for the efficient identification of mutant genes in all organisms. We identified the mutation in *mgn* as a deletion in the coding sequence of the zebrafish *microtubule actin crosslinking factor 1* (*macf1*) gene. *macf1* is a member of the highly conserved spectraplakin family of cytoskeletal linker proteins, which play diverse roles in polarized cells such as neurons, muscle cells, and epithelial cells. In *mgn* mutants, the oocyte nucleus is mislocalized; and the Balbiani body, localized mRNAs, and organelles are absent from the periphery of the oocyte, consistent with a function for *macf1* in nuclear anchoring and cortical localization. These data provide the first evidence for a role for spectraplakins in polarization of the vertebrate oocyte and egg.

## Author Summary

How the axes of the embryo are established is an important question in developmental biology. In many organisms, the axes of the embryo are established during oogenesis through the generation of a polarized egg. Very little is known regarding the mechanisms of polarity establishment and maintenance in vertebrate oocytes and eggs. We have identified a zebrafish mutant called *magellan*, which displays a defect in egg polarity. The gene disrupted in the *magellan* mutant encodes the cytoskeletal linker protein *microtubule actin crosslinking factor 1* (*macf1*). In vertebrates, it can take years to identify the molecular nature of a mutation. We used a new technique to identify the *magellan* mutation, which allowed us to rapidly isolate genomic DNA linked to the mutation and sequence it. Our results describe an important new function for *macf1* in polarizing the oocyte and egg and demonstrate the feasibility of this new technique for the efficient identification of mutations.

## Introduction

The animal-vegetal axis is the first axis to form in the vertebrate embryo; however, the cellular and genetic pathways by which it is specified are the least well understood. In lower vertebrates, such as frogs and fish, this axis forms during early oogenesis, while in mouse, it becomes apparent during oocyte maturation. The earliest morphological marker of asymmetry in the vertebrate oocyte is the Balbiani body, a transient structure composed of organelles, including mitochondria, endoplasmic reticulum (ER) and Golgi (reviewed in [1] and [2]). The Balbiani body is found in the oocytes of invertebrates such as *Drosophila* [3] and vertebrates, including *Xenopus*, mouse and human [4]–[9]. In most mammals, the molecular composition and function of the Balbiani body is unknown. In mouse, however, the Balbiani body contains Trailer hitch (Tral) protein, which has been implicated in mRNA localization and translation in *Drosophila* and in P body assembly in human cells [10], [11]. The presence of Tral in the Balbiani body of the mouse suggests a function for this structure in RNA metabolism [6].

Studies of the *Xenopus* Balbiani body have shown that in addition to ER, mitochondria and Golgi, it contains germ plasm RNAs and germinal granules, and it is thought to localize these factors to the vegetal cortex of the oocyte during early oogenesis [5], [12], [13] (reviewed in [2]). Recent studies have described a Balbiani body in zebrafish oocytes that behaves similarly to the *Xenopus* Balbiani body [14]–[17]. In both zebrafish and *Xenopus*, the Balbiani body is the first morphological marker of polarity and predicts the future animal-vegetal axis. In primary oocytes, it forms adjacent to the oocyte nucleus on the future vegetal side of the oocyte and then becomes localized to the vegetal cortex as oogenesis proceeds [5], [14]–[18]. Upon localization to the vegetal cortex, germ plasm RNAs and germinal granules are deposited and the Balbiani body disassembles.

Although a highly conserved structure, the mechanisms of Balbiani body formation, function, and disassembly have remained elusive, in large part due to a lack of genetic and molecular data. The zebrafish *bucky ball* (*buc*) gene is the only vertebrate gene known to be required for Balbiani body formation [15], [17]. *buc* encodes a novel protein, in the absence of which a Balbiani body fails to form. In *buc* mutants, vegetal RNAs are not localized, reflecting a defect in animal-vegetal polarity of the oocyte [15], [17].

We report a second zebrafish gene required for animal-vegetal polarity of the oocyte and egg. The *magellan* (*mgn*) mutant was identified based on a defect in which cytoplasm localizes around the yolk rather than at the animal pole of the egg [19]. We found that during oogenesis, *mgn* mutant oocytes display an asymmetric localization of the oocyte nucleus, a novel enlarged Balbiani body phenotype and an absence of vegetally-localized RNAs, stable microtubules, and organelles at the oocyte cortex. To identify the gene disrupted in *mgn* mutants, we positionally cloned the *mgn* gene, utilizing a novel DNA capture method to enrich for genomic DNA spanning the interval containing our candidate gene. This technique involves hybridization of region-specific oligonucleotides to long genomic DNA fragments, extension of the oligonucleotides using labeled nucleotides, capture of the labeled fragments along with the genomic DNA template and flanking regions, and finally, isolation of the captured genomic DNA, which is then processed for massively parallel sequencing. Using this technique, we identified a 31 base pair deletion in the zebrafish ortholog of *microtubule actin crosslinking factor 1* (*macf1*), a highly conserved cytoskeletal linker protein belonging to the spectraplakin family of proteins. This is one of the first examples in which genomic DNA enrichment combined with massively parallel sequencing has been used to determine the molecular identity of a gene associated with a phenotype. *macf1* function has been characterized in several types of polarized cells including epithelial cells, neurons, and muscle cells. Our analysis of the *mgn* mutant reveals a new role for *macf1* in the oocyte, providing insight into the role of spectraplakins during vertebrate oogenesis.

## Results

### Disrupted animal-vegetal polarity in the *mgn* mutant

**Figure 1:**
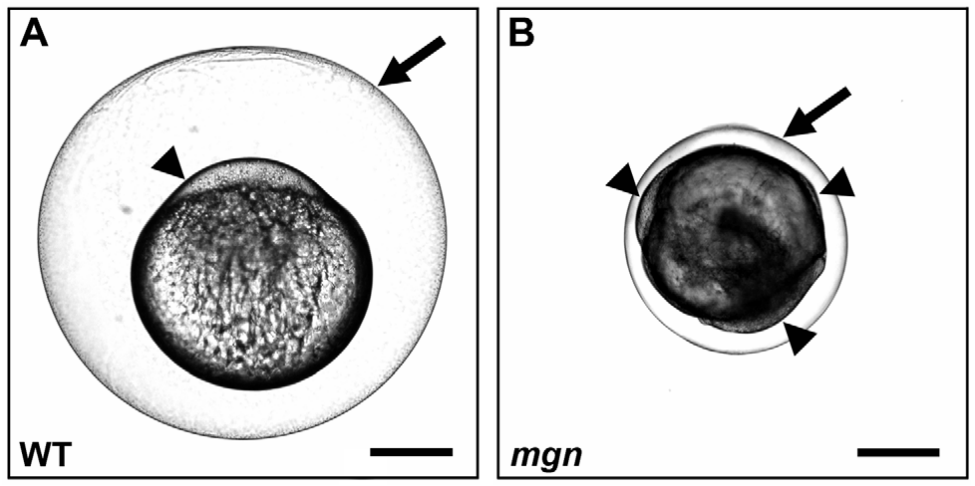
*mgn* is required for animal-vegetal polarity of the egg. Nomarski images of eggs from a wild-type (heterozygous sibling) female (A) and a *mgn* mutant female (B) one hour post activation. Eggs from *mgn* mutants exhibit cytoplasm surrounding the yolk (arrowheads) rather than restricted to the blastodisc at the animal pole as in wild-type (lateral view, animal pole up). In addition, *mgn* eggs are frequently smaller and display variable elevation of the chorion (arrow). Scale bar = 250 microns.

In zebrafish, animal-vegetal polarity of the egg becomes morphologically apparent upon egg activation. Prior to activation, the cytoplasm of the egg is intermingled with the yolk. Activation of the egg through contact with water causes two striking changes to the egg: the cortical granules fuse with the plasma membrane and exocytose their contents into the perivitelline space causing the chorion to expand; and the egg contracts, resulting in segregation of cytoplasm from the yolk to the animal pole to form the blastodisc (Figure 1A). The zebrafish *mgn* mutant was identified based on a defect in activated eggs. We found that *mgn* mutant eggs exhibit variable expansion of the chorion. In addition, we observed that following cytoplasmic segregation, cytoplasm was variably distributed around the yolk rather than being restricted to one pole (Figure 1B), suggesting a defect in animal-vegetal polarity of the egg.

### Defective oogenesis in the *mgn* mutant

**Figure 2:**
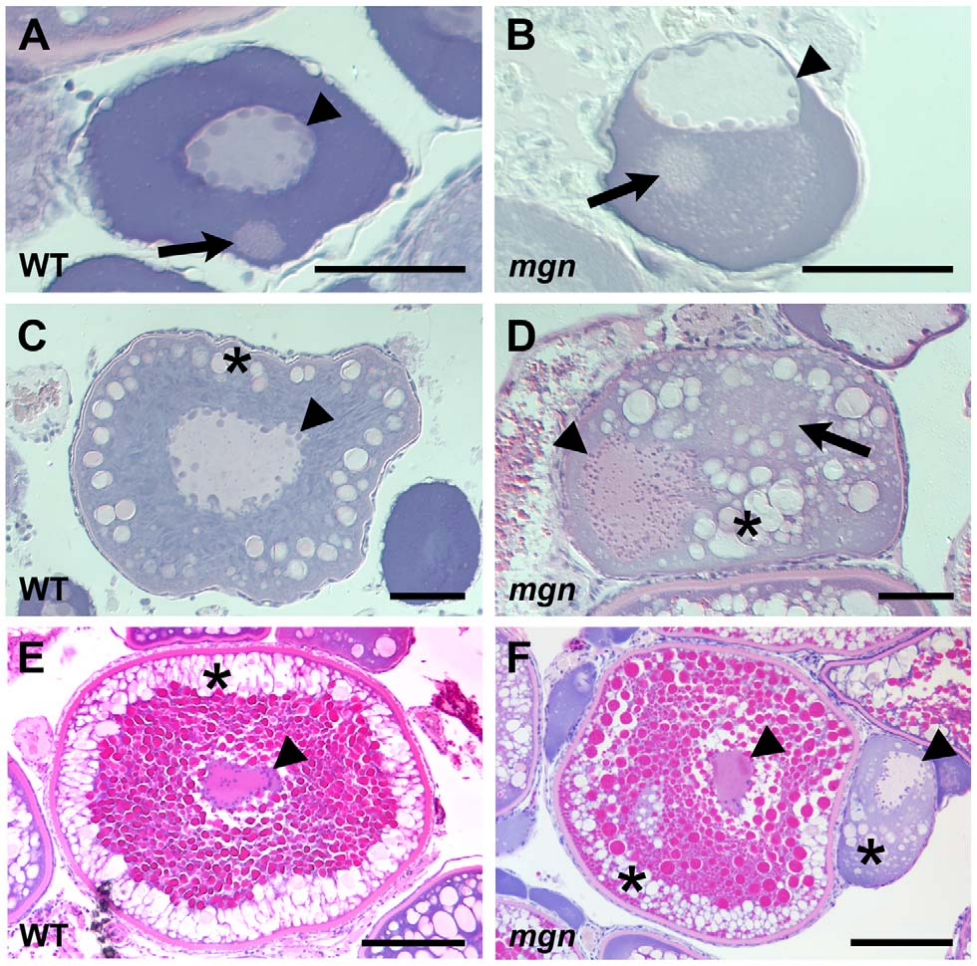
The *mgn* mutation causes defects during oogenesis. Sections of wild-type and mutant oocytes stained with hematoxylin (purple) and eosin (pink). The cytoplasm of stage I zebrafish oocytes is strongly basophilic, resulting in strong purple staining, while the mitochondria-rich Balbiani body is slightly acidophilic and stains pale pink with eosin. In wild-type mid stage I oocytes (A), the nucleus is localized at the center of the oocyte and the Balbiani body is near the future vegetal cortex. In *mgn* mutant stage I oocytes (B), the nucleus is asymmetrically localized and the Balbiani body, which frequently remains close to the nucleus, is surrounded by a region that is lightly stained with eosin. Wild-type stage II oocytes (C) have a central nucleus surrounded by cortical granules (CG). In stage II *mgn* mutant oocytes (D), the nucleus is mislocalized and CG accumulate opposite to the nucleus, in and around a faint eosin-stained area (arrow). During stage III, wild-type oocytes (E) accumulate yolk in the center of the oocyte and CG localize uniformly around the cortex, whereas stage III *mgn* mutant oocytes (F) display an uneven distribution of CG at the cortex. Also note stage II oocyte at right in (F). Arrowheads indicate oocyte nuclei; arrows indicate Balbiani body in (A,B); asterisks indicate CG. Scale bars = 50 microns (A,B), 100 microns (C,D), and 200 microns (E,F).

To determine if the *mgn* mutation causes defects during oogenesis, we examined mutant and wild-type oocytes by histology, using hematoxylin and eosin dyes to visualize structures such as the Balbiani body, cortical granules and yolk. In wild-type mid to late stage I oocytes, the nucleus was centrally located and the Balbiani body, which forms on the future vegetal side of the nucleus and translocates to the vegetal cortex, was detected either near the nucleus (n = 2/15), between the nucleus and future vegetal cortex (n = 6/15), or at the vegetal cortex (Figure 2A; n =  7/15). In contrast, the nucleus of mutant oocytes was asymmetrically localized (Figure 2B) and the Balbiani body was only infrequently found at the cortex (n = 2/20). In the majority of mutant oocytes, the Balbiani body was observed near the nucleus (Figure 2B; n = 12/20) or between the nucleus and oocyte cortex (n = 6/20). In addition, the Balbiani body of mutant oocytes was surrounded by pale pink staining that appeared speckled. This staining was not observed in wild-type oocytes.

The asymmetric positioning of the oocyte nucleus was also seen in stage II mutant oocytes, whereas in wild-type oocytes, the nucleus remained centrally located (compare Figure 2C and 2D). In addition, we found that in wild-type stage II oocytes, cortical granules were located radially around the central nucleus (Figure 2C; n = 25/25), whereas in mutant oocytes, cortical granules were asymmetrically located and were found in the middle of the oocyte, including within and around a region distinct to mutant oocytes that stains lightly with eosin (Figure 2D; n = 31/31). Stage III of oogenesis is characterized by an accumulation of yolk in the middle of the oocyte, which is thought to drive the cortical granules to the oocyte cortex (Figure 2E; n = 17/17) [20]. In stage III *mgn* mutant oocytes, as in wild-type, yolk accumulated in the oocyte and the cortical granules typically moved to the oocyte periphery. In mutant oocytes, however, there was a variable and uneven distribution of cortical granules around the cortex and a small number of cortical granules remained within the yolk (Figure 2F; n = 22/23), likely due to their abnormal localization during stage II. These defects in cortical granule localization may result in the variable chorion expansion defect in *mgn* mutants (Figure 1).

### Defective germ plasm mRNA localization in *mgn* mutants

**Figure 3:**
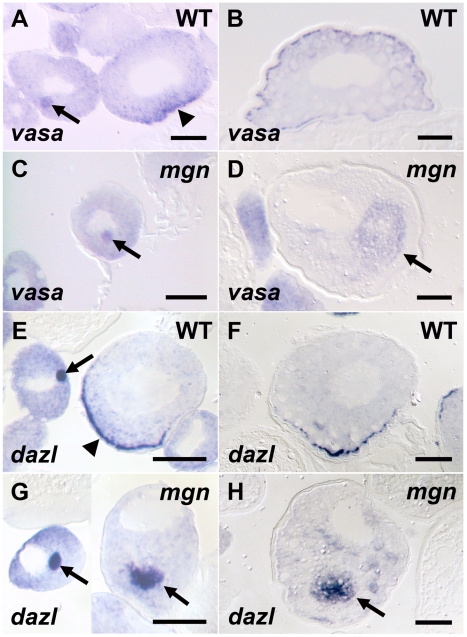
Germ plasm RNAs fail to localize to the cortex of *mgn* mutant oocytes. *vasa* (A–D) and *dazl* (E–H) *in situ* hybridization to wild-type and *mgn* mutant oocytes. *vasa* mRNA is found in the Balbiani body in early stage I wild-type (heterozygous) oocytes (A, arrow), transiently localizes to the vegetal cortex in late stage I (A, arrowhead) and then localizes all around the cortex during stage II (B). In *mgn* mutant oocytes, *vasa* mRNA is found in the Balbiani body in early stage I oocytes (C, arrow) and is then found ectopically localized to the middle of the oocyte during stage II (D, arrow). In early stage I wild-type oocytes, *dazl* mRNA is found in the Balbiani body (E, arrow) and then localizes to the vegetal cortex during late stage I (E, arrowhead) and stage II (F). *dazl* mRNA localizes to the Balbiani body of early stage I *mgn* mutant oocytes (G, left oocyte, arrow) and is found ectopically localized to the middle of the oocyte during late stage I and stage II (G, right oocyte and H, respectively, arrow). Scale bars = 50 microns.

In zebrafish, as in *Xenopus*, the Balbiani body contains germ plasm components that include germ plasm RNAs and germinal granules [14], [15], [17]. Studies in *Xenopus* indicate that the Balbiani body is required for localization of mRNAs to the vegetal cortex [12], [13], (reviewed in [2]) and recent work in zebrafish shows that distinct localization elements within the 3′UTR of *dazl* mRNA localize it to the Balbiani body and vegetal cortex of the oocyte [14]. To investigate the function of the Balbiani body in *mgn* mutant oocytes, we examined the localization of *dazl* and a second conserved germ plasm mRNA, *vasa*. In wild-type oocytes, *vasa* mRNA is localized to the Balbiani body during early stage I of oogenesis, transiently localizes to the vegetal cortex during late stage I, and then becomes localized around the cortex of the oocyte during stage II (Figure 3A and 3B). In *mgn* mutant oocytes, *vasa* mRNA localized to the Balbiani body during early stage I, but it was predominantly localized to the middle of the oocyte during late stage I and stage II (Figure 3C and 3D; n = 15/16). In wild-type oocytes, *dazl* mRNA is localized to the Balbiani body during early stage I and then becomes localized to the vegetal cortex during late stage I where it remains during stage II (Figure 3E and 3F). In mutant oocytes, *dazl* mRNA initially localized to the Balbiani body during stage I, and like *vasa* mRNA, was predominantly found in the middle of the oocyte in late stage I and stage II, rather than localized to the vegetal cortex (Figure 3G and 3H; n = 24/25). These results show that germ plasm mRNAs localize to the Balbiani body of mutant oocytes during early stage I and that Mgn is required for the subsequent localization of the Balbiani body and associated RNAs to the vegetal cortex during late stage I. In addition, the persistence of both *vasa* and *dazl* mRNA in a central domain of stage II mutant oocytes may reflect a failure of the Balbiani body to disassemble at the end of stage I of oogenesis.

### Identification of *mgn* gene by genomic DNA capture and massively parallel sequencing

**Figure 4:**
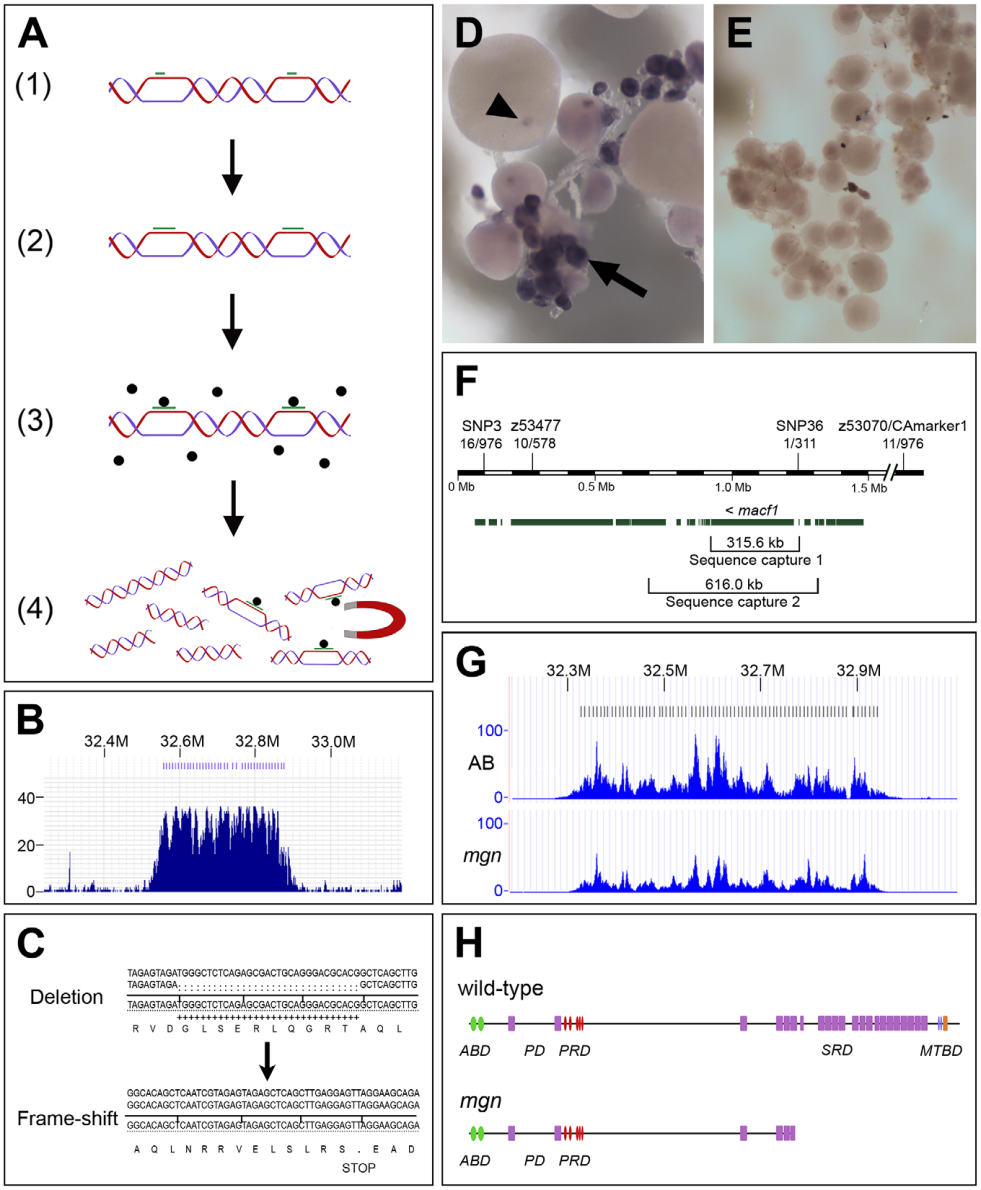
The molecular lesion in *mgn* mutants was identified by a combination of genomic sequence capture and massively parallel sequencing. (A) Schematic diagram depicting sequence capture method, which involves: 1) Hybridization of oligos (green bars) to long genomic DNA fragments at approximately 8 kb intervals; 2) Extension of oligos with biotinylated nucleotides; 3) Capture of targeted DNA with magnetic streptavidin-coated beads; 4) Extraction of captured DNA. (B) Histogram showing read depth for the 319.8 kb sequence capture region within a 0.9 Mb genomic interval. Increased read depth in the targeted region reflects the 243-fold average enrichment of the captured sequence (x-axis = chromosomal region containing targeted interval; y-axis = number of reads aligned to the region). Oligo binding positions are depicted as blue bars at the top of the figure. (C) 31 base pair deletion in *macf1* in *mgn* mutants that results in a frame-shift and premature stop codon. Sequence at top shows the deletion from a heterozygous sibling; sequence at bottom shows the frame-shift in a homozygous mutant. (D–E) *In situ* hybridization of *macf1* probe to wild-type (D) and *mgn* mutant (E) oocytes. *macf1* is expressed in stage I wild-type oocytes (D, arrow) and is localized to a restricted domain during mid to late oogenesis (D, arrowhead). Levels of *macf1* transcript are reduced in *mgn* mutant oocytes (E). (F) Genomic interval showing SSLP and SNP markers used for mapping, candidate genes, and sequence capture regions. *macf1* is transcribed from the reverse strand. Hash marks indicate 0.5 Mb of sequence not shown. Annotation based on Ensembl Zv7/danRer5 with additional annotation information from Zv8/danRer6. (G) Histograms showing read depth for the 616 kb sequence capture region within a 0.9 Mb interval. The increased read depth within the targeted region reflects the 72- and 43-fold enrichment of captured sequence from the AB and *mgn* mutant (TU) genomic DNA, respectively. Oligo binding positions are depicted as bars at the top of the figure. (H) Predicted protein structure of zebrafish Macf1 (Pfam database) and expected truncation in the spectraplakin domain. Actin binding domain (ABD), Plakin domain (PD), Plakin repeat domain (PRD), Spectraplakin repeat domain (SRD), Microtubule binding domain (MTBD). Green = Calponin homology domain; purple = Spectrin domain; red = plakin domain; blue = EF hand domain; orange = GAS2 domain.

Using SSLP (simple sequence length polymorphism) markers in bulk segregant analysis, followed by fine mapping of homozygous mutant versus heterozygous sibling females using SSLP and SNP (single nucleotide polymorphism) markers, we localized the *mgn* mutation to a 2.02 Mb interval (Figure 4F) based on the Sanger Institute Ensembl zebrafish genome sequence assembly (Zv7/danRer5). Based on data from 976 meioses and 27 recombination events, we then calculated that the molecular lesion would be in the zebrafish *macf1* gene. To date, a full-length zebrafish *macf1* transcript has not been sequenced, however, the longest predicted zebrafish *macf1* transcript is 23,975 base pairs (bp) (NCBI RefSeq accession: XM_001920059.1). Additionally, while *macf1* is highly conserved, the frequency of multiple, alternatively spliced isoforms in other organisms [21] suggested that prediction of ovarian transcript sequences would be difficult. We therefore chose to utilize increasingly affordable next-generation sequencing technologies to sequence the entire 298,606 bp predicted *macf1* genomic interval (Zv7/danRer5, Ensembl 52 genebuild) as well as approximately 17 kb of sequence 5′ to the predicted gene. To do this economically, we had to enrich for genomic sequence specific to our target *macf1* region. This was accomplished using a genomic sequence capture and release method. This method involves designing 22–27mer long region-specific oligonucleotides (oligos) that are hybridized to genomic DNA, extended using biotinylated nucleotides, and then affinity purified using streptavidin coated magnetic beads to capture the genomic template DNA (modification of method in [22]). The captured DNA is then dissociated from the beads, sheared, and cloned into a library for massively parallel sequencing (Figure 4A).

We designed oligos approximately every 8 kb spanning a 319.8 kb region, performed the capture and sequencing, and then analyzed a 315.6 kb region that included all of *macf1* and ∼17 kb of sequence 5′ to the gene. We obtained a 243-fold average enrichment for this region, as determined from the sequence analysis. The sequencing results also showed a 31-fold average read depth for unique sequence, covering 99.9% of our targeted region (Figure 4B). Data were obtained for the entire predicted coding sequence with the exception of three small gaps. The genomic intervals surrounding these gaps were then amplified by PCR and sequenced by Sanger sequencing. A four bp and 12 bp gap were not confirmed by Sanger sequencing and no base changes were found in these intervals. Sanger sequencing did, however, confirm a gap of 31 bp. The 31 bp deletion causes a frame-shift at amino acid codon 5315 of the longest predicted zebrafish Macf1 protein isoform (NCBI RefSeq accession: XP_001920094.1), resulting in a premature stop codon and subsequent truncation of the predicted protein (Figure 4C). One additional base change in predicted coding sequence was found by massively parallel sequencing but was not confirmed by Sanger sequencing.

Based on the *mgn* mutant phenotype, we expected *macf1* to be expressed during stage I of oogenesis. *In situ* hybridization experiments showed that, consistent with a function during early oogenesis, *macf1* mRNA is expressed in stage I oocytes and additionally, has a restricted localization pattern during later oogenesis (Figure 4D). In contrast, *in situ* hybridization analysis showed that in *mgn* mutant oocytes, *macf1* mRNA levels appeared to be significantly reduced (Figure 4E). This suggests that the transcript is subject to nonsense-mediated decay and is consistent with the presence of a premature stop codon in *macf1* caused by the deletion in *mgn* mutants.

To confirm that the deletion in *macf1* is the only change in coding sequence in the interval to which the *mgn* mutation maps, we further narrowed the interval and then sequenced the remaining candidate genes. We initially attempted to identify SNPs to narrow the interval by PCR amplification and sequencing of non-coding regions, but we were unsuccessful in identifying polymorphisms. Therefore, we performed a second sequence capture experiment with wild-type and mutant fish from a new map cross to attempt to more efficiently identify any rare SNPs in the region and examine additional genes in the interval. For the second sequence capture, we designed oligos to a 613.9 kb region that consisted of the *macf1* gene plus approximately 86 kb of sequence 5′ to *macf1* and 230 kb of sequence 3′ to *macf1* (Figure 4F).

We massively parallel sequenced the captured genomic DNA from a wild-type (AB strain) fish and a mutant (TU strain) fish and analyzed 616 kb of targeted sequence and surrounding sequence that was captured. For the wild-type sample, we obtained a 72-fold average enrichment and a 29-fold average read depth for unique sequence, covering 94% of our targeted region (Figure 4G). For the mutant sample, we obtained a 43-fold average enrichment and a 17-fold average read depth for unique sequence, covering 97% of our targeted region (Figure 4G). Using these data, we identified a SNP (called SNP36) 14.35 kb from the 5′ end of *macf1*. No genes are known or predicted to exist in this 14.35 kb region. We were unable to identify useful SNPs 3′ to the *macf1* gene using the sequence capture data; therefore, we used a previously identified SSLP marker (z53477) to narrow the interval 3′ to *macf1*. We identified one recombinant fish from 311 meioses at SNP36 and 10 recombinants from 578 meioses at z53477.

The interval between z53477 and SNP36 contains part of *si:dkey-190l1.2* (similar to vertebrate gamma-aminobutyric acid B receptor, 2), as well as the full sequence of 13 genes. Ten genes including *macf1* are present in the second sequence capture interval (*sla1 (Src-like-adaptor 1)*, *wisp1a* (novel protein similar to vertebrate WNT1 inducible signaling pathway protein 1), *ndrg1* (*N-myc downstream regulated gene 1*), *si:rp71–45k5.2* (novel protein), *zgc:113424* (novel forkhead domain-containing protein), *si:rp71–45k5.3* (novel protein), *si:rp71–45k5.4* (novel protein similar to vertebrate proteasome subunit, alpha type 2), *zgc:91910* (novel protein), *bmp8 (bone morphogenetic protein 8)* and *macf1*). Examination of the coding and predicted coding sequences revealed that there were no sequence changes in any of the genes in the interval. Three genes in the *mgn* interval that were not included in the captured region were cloned from ovarian cDNA (*si:dkey-190l1.1* (*elongator complex protein 2*), *si:ch211–254e15.2* (*UPF0436 protein C9orf6* homolog), *si:ch211–254e15.1* (novel protein similar to vertebrate catenin, alpha-like 1)) and were sequenced by Sanger sequencing. No sequence changes were found in the predicted coding regions of these genes. Based on these data, we conclude that the *mgn* mutant phenotype is caused by the deletion in *macf1*.

*macf1* belongs to the highly conserved spectraplakin family of proteins. Spectraplakins are multifunctional cytoskeletal linker proteins characterized by an N-terminal actin binding domain consisting of Calponin homology domains, a globular plakin domain that mediates interactions with various types of cell junctions, a plakin repeat domain that can interact with intermediate filaments, a spectrin repeat domain that allows dimerization, and finally a C-terminal microtubule binding domain that consists of an EF-hand and GAS2 domain [23], [24]. The *mgn* deletion creates a stop codon in the spectrin repeat domain that is predicted to result in a loss of part of the spectrin domain and all of the microtubule binding domain (Figure 4H). Thus, the truncated protein is not expected to bind to the microtubule cytoskeleton.

### Loss of peripheral localization of stable microtubules

**Figure 5:**
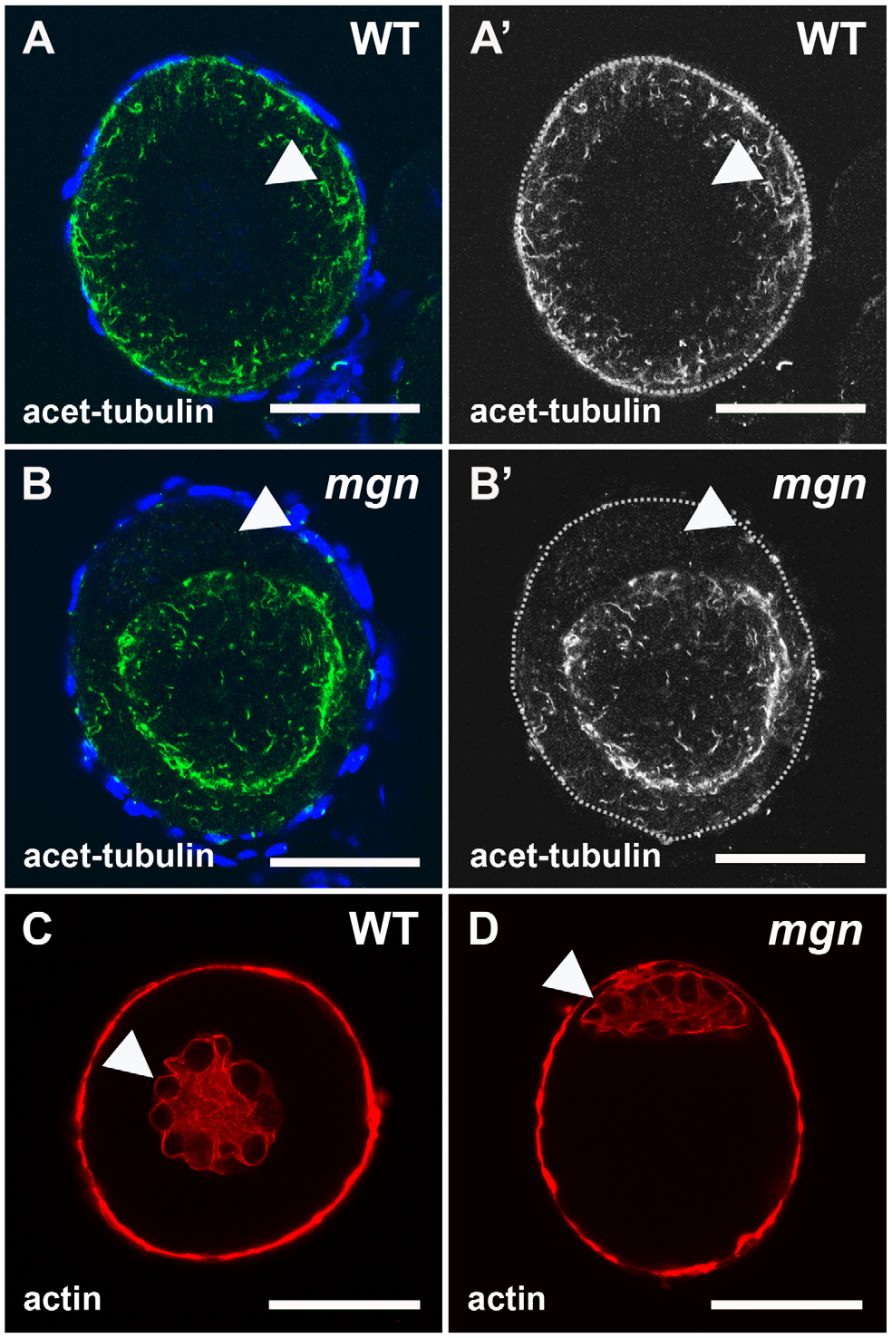
Localization of stable microtubules but not actin is disrupted in *mgn* mutant oocytes. Stage I oocytes stained with an antibody to acetylated tubulin to label stable microtubules (A,B) or rhodamine phalloidin to label actin cytoskeleton (C,D). In wild-type stage I oocytes, acetylated microtubules are uniformly distributed throughout the oocyte (A,A′), whereas in *mgn* mutant oocytes, acetylated microtubules are largely absent from peripheral regions of the oocyte (B,B′). DAPI staining around oocytes labels nuclei of surrounding somatic follicle cells. Green = acetyated tubulin; blue = DAPI. In both wild-type (C) and *mgn* mutant oocytes (D), actin is localized to the nucleus and at the cortex. Arrowheads indicate oocyte nuclei. Red = phalloidin. Scale bars = 25 microns. All images are single optical sections.

It is well established that spectraplakins such as *macf1* are able to interact with both the actin and microtubule cytoskeletons (reviewed in [21], [24]). To determine if the cytoskeleton is affected in *mgn* mutant oocytes, we first used an antibody to acetylated tubulin to visualize stable microtubules. We found that in wild-type stage I oocytes, stable microtubules were present throughout the oocyte but did not appear to be attached to a microtubule organizing center (MTOC) (Figure 5A and 5A′; n = 13/13). In zebrafish as in other organisms, the centrosome, which serves as an MTOC, is inactivated early in oogenesis. Stable microtubules were also present in *mgn* mutant oocytes, but their presence was significantly reduced at the periphery of the oocyte (Figure 5B and 5B′; n = 23/24). We next examined the actin cytoskeleton in wild-type and *mgn* mutant oocytes. In both wild-type and mutant oocytes, we found actin filaments localized to the nucleus and at the oocyte cortex (Figure 5C and 5D, n = 15 wild-type and n = 13 mutant oocytes). Thus, the mutation in *macf1* causes a loss of stable microtubule localization to the periphery of the oocyte, but it does not appear to affect the actin cytoskeleton. These results suggest that, consistent with loss of the predicted Macf1 microtubule binding domain, the defects seen in *mgn* mutants may be caused by a loss of tethering of stable microtubules to the oocyte cortex.

### The *mgn* mutation affects organelle localization and Balbiani body size

**Figure 6:**
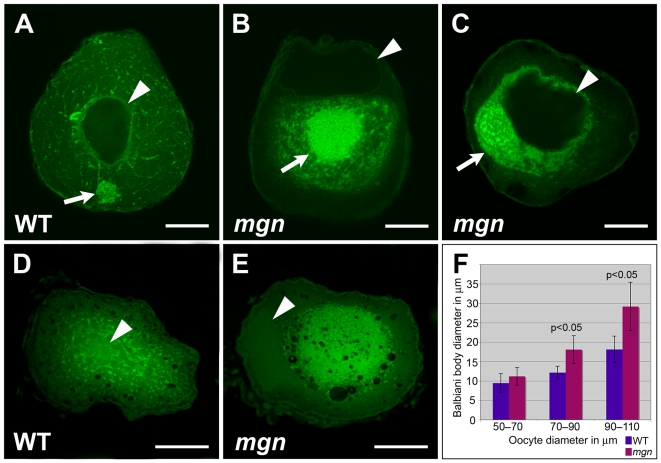
*mgn* mutant oocytes exhibit an enlarged Balbiani body and absence of mitochondria and ER from the periphery of the oocyte. Oocytes stained with DiOC_6_ to label ER and mitochondria (A–E). In a wild-type mid stage I oocyte, the nucleus is in the middle of the oocyte (arrowhead) and the Balbiani body is at the future vegetal side of the oocyte (A, arrow). (B,C) Mutant oocytes exhibit an enlarged Balbiani body (arrows), and an absence of ER, mitochondria and the Balbiani body from the periphery. Images are single optical sections. In stage II wild-type oocytes (D), ER and mitochondria are localized throughout the oocyte, whereas in *mgn* mutant oocytes (E), ER and mitochondria are concentrated in the middle of the oocyte and are absent from peripheral regions. Images are single optical sections of 0.5 micron oocyte sections. (F) Bar graph depicting size of Balbiani body (Bb) during stage I of oogenesis. 50–70 micron oocytes, n = 10 wild-type and 10 mutant oocytes; 70–90 micron oocytes, n = 15 wild-type and 15 mutant oocytes; 90–110 micron oocytes, n = 15 wild-type and 15 mutant oocytes. Arrowheads indicate nuclei. (A–C) scale bars = 25 microns. (D,E) scale bars = 100 microns.

Given the defect in cortical localization of RNAs and stable microtubules, we investigated if *mgn* mutant oocytes also exhibit defects in the localization of organelles by labeling ER and mitochondria with the membrane dye, DiOC_6_. In addition to cytoplasmic ER and mitochondria, DiOC_6_ labels the aggregate of ER and mitochondria that, along with germ plasm mRNAs and other organelles, composes the Balbiani body (reviewed in [1], [2]). DiOC_6_ staining revealed that in wild-type oocytes, ER and mitochondria are found throughout the oocyte and in addition, are concentrated within the Balbiani body, which translocates from the nucleus to the oocyte cortex during mid to late stage I (Figure 6A, n = 80/80). In mutant oocytes, ER and mitochondria, as well as the Balbiani body itself, are typically absent from the periphery of the oocyte (Figure 6B and 6C, n = 46/64). In many oocytes, ER and mitochondria were tightly concentrated around the nucleus of the oocyte, as well as absent from the periphery (Figure 6C; n = 24/64). The defect in the peripheral localization of ER and mitochondria persists into stage II of oogenesis when in wild-type oocytes, the Balbiani body has disassembled and ER and mitochondria are localized throughout the oocyte (Figure 6D). In *mgn* mutants, DiOC_6_ staining was concentrated in the middle of stage II mutant oocytes (Figure 6E), consistent with a function for *macf1* in the localization of ER and mitochondria to peripheral regions of the oocyte. The lack of ER and mitochondria localization to the periphery of *mgn* mutant oocytes is strikingly similar to the absence of acetylated tubulin in these regions (Figure 5A and 5B).

**Figure 7:**
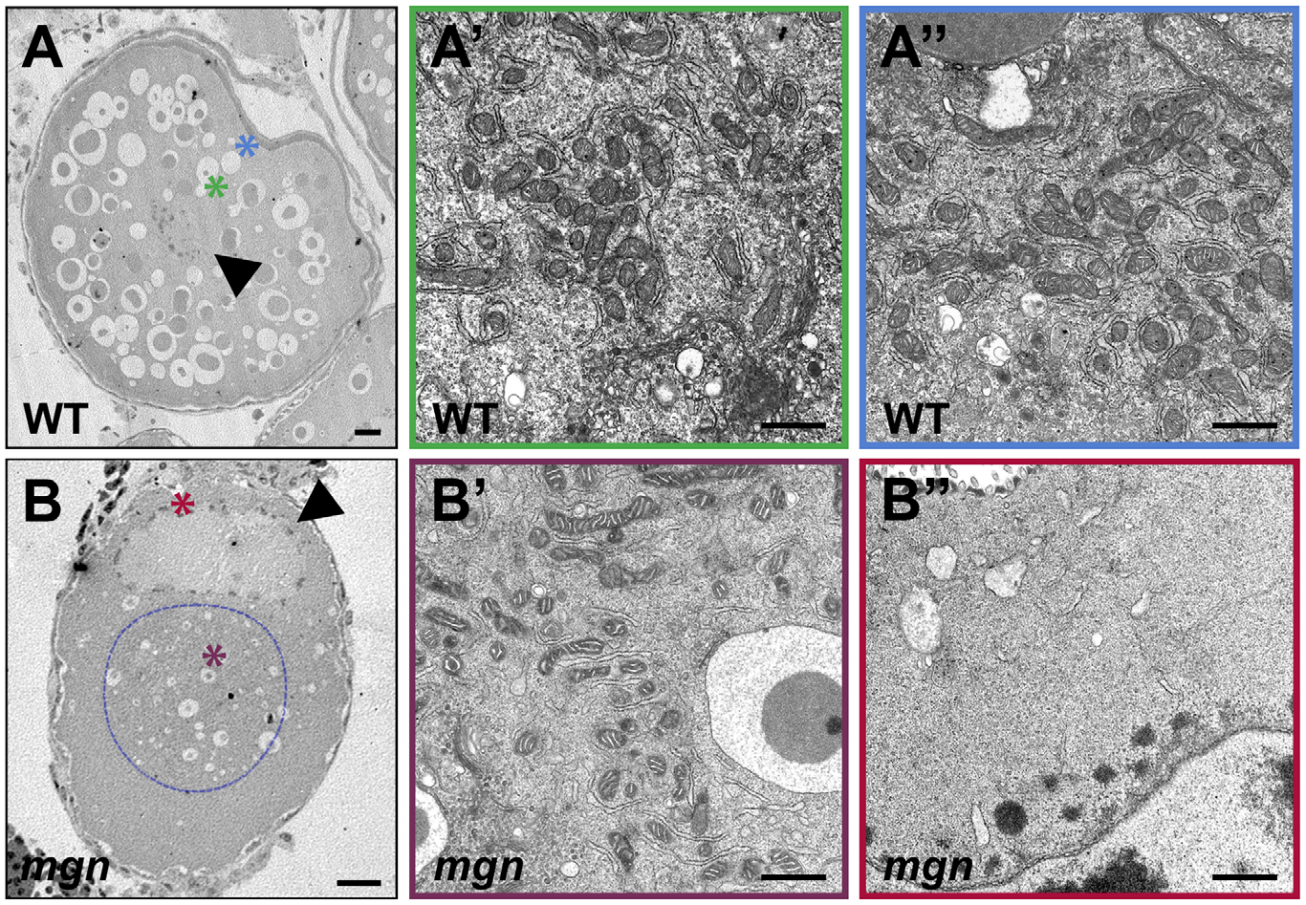
ER and mitochondria are absent from the periphery of stage II *mgn* mutant oocytes. Transmission electron micrographs of stage II wild-type (A–A″) and *mgn* mutant (B–B″) oocytes. ER and mitochondria are distributed throughout wild-type stage II oocytes (A–A″) but are absent from the periphery of stage II *mgn* mutant oocytes (B–B″). Arrowheads indicate oocyte nuclei. Positions of regions shown at high magnification (A′, A″, B′ and B″) are indicated by the respective colored asterisks in A and B. Dashed blue line outlines the electron dense region in which most of the ER and mitochondria are located. Scale bars = 20 microns (A, B) and 1 microns (A′, A″, B′ and B″).

To confirm the absence of ER and mitochondria at the oocyte periphery observed with DiOC_6_ staining, we examined stage II oocytes by transmission electron microscopy (TEM). TEM revealed a uniform distribution of ER and mitochondria throughout wild-type oocytes (compare cytoplasmic and peripheral domains in Figure 7A′ and 7A″; n = 4). In *mgn* mutant oocytes, the central cytoplasmic domain was similar to that of wild-type oocytes (Figure 7B′; n = 7); however, in peripheral regions, very little ER and no mitochondria were present (Figure 7B″). These TEM results are consistent with our observations from the DiOC_6_ staining and further support a role for *macf1* in the peripheral localization of organelles.

In addition to the frequent loss of cortical Balbiani body localization, DiOC_6_ staining revealed a difference in Balbiani body size in mutant compared to wild-type oocytes. We found that in early stage I mutant oocytes, the Balbiani body was comparable in size to that of wild-type oocytes (Figure 6F; 50–70 micron diameter); however, in mid to late stage I oocytes (70–110 micron diameter) the Balbiani body of mutant oocytes was significantly larger than in wild-type oocytes (Figure 6F, average Balbiani body size = 12 microns versus 18 microns in 70–90 micron wild-type and mutant oocytes, respectively, and 18 microns versus 29 microns in 90–110 micron wild-type and mutant oocytes, respectively). Thus, the Bb is similarly sized in early stage I *mgn* mutant and wild-type oocytes but becomes abnormally large beginning in mid stage I of oogenesis in *mgn* mutants.

The asymmetric localization of the nucleus in mutant stage I and II oocytes that we observed by histology was also apparent in DiOC_6_ stained oocytes (Figure 6B). In 70–90 micron wild-type oocytes, the average distance between the centroid of the nucleus and the centroid of the oocyte was 6.3 microns (n = 15), whereas in 70–90 micron mutant oocytes, it was 12 microns (n = 13). Thus, there is a significant (p<0.05) difference in the position of the nucleus in mutant mid stage I oocytes. It is not yet clear if the mislocalized nucleus causes the defect in Balbiani body size, is a consequence of it, or is an independent defect of *mgn* mutant oocytes.

## Discussion

The mechanisms guiding the establishment and maintenance of polarity in the vertebrate oocyte are not well understood. Here we have identified a novel function for *macf1* in formation of the animal-vegetal axis during zebrafish oogenesis. *macf1* belongs to the spectraplakin family of cytoskeletal linker proteins, which are highly conserved amongst species. Mutations in the *Drosophila* homolog of *macf1*, *short stop* (*shot*), were first identified in screens for neuromuscular specificity and axon guidance [25], [26] and for integrin-mediated adhesion [27]–[29]. Subsequent work has defined several additional roles for *shot* in processes that include oocyte determination and tracheal cell fusion [30], [31]. The diverse functions of *shot* have been attributed to the presence of multiple isoforms, and recent work has focused on defining tissue-specific domain requirements [32]. The function of *macf1* in vertebrates has largely been characterized in cell culture experiments, since the mouse *macf1* knockout results in embryonic lethality [33], [34]. As in *Drosophila*, vertebrate *macf1* has diverse functions. In Hela cells, for example, *macf1* is required for the cortical localization of CLASP2, a microtubule tip binding protein [35]. During mouse embryogenesis, *macf1* is required for Wnt signaling, where it functions in translocating Axin to the cell membrane [34]. Our isolation of the zebrafish *mgn* mutant provides a unique opportunity to now study the role of *macf1* during vertebrate oogenesis.

We found that *mgn* is required during zebrafish oogenesis for formation of the animal-vegetal axis of the oocyte and egg. The only other vertebrate gene known to be required for this process is the zebrafish *bucky ball* (*buc*) gene, which encodes a novel protein [15], [17]. In *buc* mutants, a Balbiani body does not form and RNAs are mislocalized, leading to a defect in animal-vegetal polarity of the oocyte and egg [15], [17]. Similar to *buc* mutants, *mgn* mutant females produce eggs that display abnormal animal-vegetal polarity. In *mgn* mutant oocytes, however, a Balbiani body forms during early stage I as in wild-type oocytes, but it becomes abnormally large and typically does not localize to the oocyte cortex during mid stage I as in wild-type. Interestingly, a null mutation in Kinesin heavy chain or P-element insertions in the kinesin-associated mitochondrial adaptor, *milton*, which cause its overexpression, result in overgrowth of the Balbiani body in the *Drosophila* ovary [36]. Although similar to *mgn* mutants, the Balbiani body defects in these *milton* mutants affect the localization of mitochondria to the Balbiani body without disrupting RNA localization and are largely resolved during late oogenesis when the microtubule cytoskeleton reorganizes [36]. Thus, *mgn* is the only gene known to be required for regulating the size of the Balbiani body, as well as for localization of the Balbiani body and its associated mRNAs to the oocyte cortex. Macf1 may be directly required for these processes through binding to cytoskeletal elements within the Balbiani body. Alternatively, misregulation of Balbiani body size and localization in *mgn* mutants may be caused by a disruption of the stable microtubule cytoskeleton that prevents localization of the Balbiani body to the vegetal cortex of the oocyte.

Analysis of mid stage I *mgn* mutant oocytes revealed an asymmetric localization of the nucleus that is not seen in wild-type oocytes at this stage and which persists into stage II. It is possible that this mislocalization of the oocyte nucleus is a consequence of the Balbiani body enlargement; however, it is also plausible that the nuclear mislocalization represents an independent defect in nuclear anchoring. Establishment and maintenance of a centralized nucleus is an active process, and it is well established that actin filaments, microtubules, and intermediate filaments play roles in nuclear migration and anchoring in various cell types (reviewed in [37]–[39]). Nuclear anchoring is mediated by a family of proteins, all of which contain KASH (Klarsicht, ANC-1, Syne homology) domains at their C-termini. The KASH domains target the proteins to the outer nuclear envelope, while the N-termini of these proteins tether them to the cytoskeleton (reviewed in [40], [41]). Interestingly, the KASH domain of Nesprin-3a interacts with the actin-binding domain of MACF1 in coimmunoprecipitation experiments *in vitro* [42]. It has recently been reported that vertebrate Macf1 isoform-3 localizes to the outer nuclear envelope and that the N-terminus associates with Nesprin-3 in COS-7 cells [43]. These data suggest that Macf1 may play a role in tethering the nucleus to the microtubule cytoskeleton. Such a function for Macf1 would be consistent with the defect in nuclear positioning that we observe in *mgn* mutant oocytes.

In addition to the abnormally large Balbiani body and the nuclear positioning defect, DiOC_6_ staining and TEM revealed that the cytoplasmic ER and mitochondria that surround the Balbiani body were absent from the periphery of the oocyte. It has long been recognized that microtubules are essential for establishing and maintaining the structure of the ER (reviewed in [44]) and for localizing mitochondria to various cellular domains (reviewed in [45], [46]). Knockdown of the Kinesin-binding protein Kinectin in cultured cells results in a collapse of ER and mitochondria to the center of the cell [47], demonstrating the involvement of motor proteins in the maintenance of ER architecture and mitochondrial localization. In addition, in cultured mouse cells mutant for the conventional kinesin heavy chain, *kif5b*, mitochondria display a perinuclear distribution and an abnormal absence from the cell periphery [48]. Several non-motor proteins have also been implicated in mediating ER-microtubule interactions (reviewed in [44], [49]). Climp-63, for example, is an integral ER membrane protein that binds to microtubules, anchoring the ER to the cytoskeleton. Overexpression of a mutant Climp-63 lacking the microtubule binding domain results in displacement of endogenous Climp-63 and clustering of ER around the nucleus [50]. These phenotypes are similar to those of *mgn* mutant oocytes, in which ER and mitochondria are frequently concentrated around the nucleus and are absent from the periphery. This suggests that Macf1 may be required to maintain ER architecture and to localize mitochondria in the oocyte by positioning stable microtubules at the oocyte periphery. Alternatively, Macf1 may bind directly to ER, mitochondria and microtubules.

We found that *mgn* is required to localize stable microtubules to peripheral regions in zebrafish oocytes. Microtubule-cortex interactions are mediated by a growing class of microtubule-associated proteins called microtubule plus-end-tracking proteins (+TIPS). +TIPS mediate cortical interactions through association of microtubule ends with the cortical actin cytoskeleton and with the plasma membrane (reviewed in [51], [52]). Macf1 (Acf7) has been identified as a +TIP in mouse endodermal cells where it functions to coordinate the actin and microtubule cytoskeletons in the cytoplasm and at the cell cortex and to maintain polarity in response to wound healing [33]. In *Drosophila* tendon cells, Macf1 (Shot) organizes the microtubule network at the muscle-tendon junction by forming a complex with the +TIPs EB1 and APC [53], which regulate cortical targeting of microtubules (reviewed in [51], [54]). Recent studies have shown that Macf proteins bind to EB1 directly and exhibit plus end tracking *in vivo* [55]. These data are consistent with a function for Macf1 in cortical localization of microtubules. Together, the loss of stable microtubules at the oocyte periphery and the absence of peripheral ER and mitochondria in *mgn* mutant oocytes suggest that in the zebrafish oocyte, ER and mitochondria associate with stable microtubules and that Macf1 may function to tether the stable microtubule cytoskeleton to the cortical actin cytoskeleton. In the absence of functional Macf1, the microtubule cytoskeleton is disrupted and the ER and mitochondria collapse into the middle of the cell.

To identify the molecular lesion associated with the *mgn* phenotype, we identified a candidate gene using positional cloning methods and then used a novel targeted sequence capture technique combined with massively parallel sequencing to sequence the candidate gene and exclude nine neighboring genes in the interval. Identification of mutations by traditional positional cloning techniques is time consuming and can be hindered by a variety of factors including limited genome annotation and complex genetic loci, which make prediction of transcripts difficult. As a result, there is increasing interest in utilizing next-generation sequencing technologies to identify mutations by sequencing large regions of genomic DNA. It was recently demonstrated that a point mutation in the *encore* gene in *Drosophila* could be identified by whole genome resequencing [56]. Although extremely efficient, this method would be prohibitively costly in an organism with a large genome, such as zebrafish or mouse. A more cost-effective approach was recently used to identify a mutation in the mouse *Megf8* gene [57]. This approach utilized positional cloning techniques to identify a 2.2 Mb interval containing the mutation. The authors then constructed a BAC contig spanning this region and sequenced 15 BACs with massively parallel sequencing to identify a point mutation in *Megf8*. While this method is effective and more cost-efficient than whole genome resequencing, the construction of BAC contigs remains labor intensive and time consuming.

We sought to utilize an efficient and cost-effective method for region-specific enrichment of genomic DNA to allow for massively parallel resequencing of targeted genomic intervals. In recent years, several methods have been developed for genomic enrichment [58]–[66]. A method for in-solution hybrid selection was recently reported based on hybridizing long biotinylated RNA sequences to DNA that has been randomly sheared and amplified [66]. Oligonucleotide tiling arrays were recently used to target and identify mutations in the mouse *kit* gene, human *neurofibromin* gene, and in seven human autosomal recessive ataxia-associated genes [61]–[63]. In addition, array-based genomic sequence capture of a 40 Mb linkage interval combined with massively parallel sequencing was used to identify *TSPAN12* as the mutated gene in patients with familial exudative vitreoretinopathy [64]. Array based capture of a 2.9 Mb interval was also used to identify mutations in *taperin (C9orf75)* as the cause of nonsyndromic deafness DFNB79 [65].

We used an in-solution method to capture a targeted region based on enzymatic extension of a hybridized oligo to a targeted genomic region using biotin-labeled nucleotides based on a previously established method [22]. This region-specific extraction (RSE) method reliably and efficiently extracts long genomic DNA fragments with streptavidin-coated microparticles [22], [73]. Because RSE does not require shearing the genomic DNA prior to capture, as the previously described methods do, it retrieves large regions of genomic DNA with a very small number of oligos (each spaced about 8 kb apart), making experimental design simple and cost-efficient. Using this method, we were able to achieve 99.9% coverage of our 300 kb targeted region and 97% of our 600 kb targeted region, and we demonstrated that this method is effective in the identification of significantly larger deletions than had previously been shown [61]. Based on the excellent read depth and coverage that we obtained, our method is equally effective at identifying point mutations and SNPs, as we did here.

We used a combination of targeted genomic DNA capture and massively parallel sequencing to identify a deletion in zebrafish *macf1* that affects Balbiani body function and causes a defect in oocyte polarity. Although conditional targeting of mouse *macf1* (*acf7)* in skin epidermis [67] and brain [68] has recently been reported, the function of vertebrate *macf1* has primarily been studied in cell culture due to the lethality of the *macf1 (acf7)* knockout in mice. Until our identification of the *mgn* mutant, a requirement for vertebrate *macf1* had not been examined in the oocyte. We have shown that *macf1* is required for cortical localization of the Balbiani body, RNAs, microtubules, and organelles in the zebrafish oocyte and that disruption of *macf1* function affects polarity of the oocyte. Further studies will be required to define the molecular function of *macf1* during oogenesis and will be particularly valuable for understanding the mechanism by which cell polarity is regulated in the ovary.

## Materials and Methods

### Ethics statement

The animal work in this study was approved by the Institutional Review Board of the University of Pennsylvania School of Medicine.

### Phenotypic characterization

Phenotype characterization was carried out using the *p6cv* allele of *mgn* [19]. Oocytes were staged according to Selman et al. [20].

### Histology and *in situ* hybridization

Ovaries were dissected from euthanized females and fixed overnight at 4°C in 4% paraformaldehyde. Following washes in PBS, ovaries were processed for histology or dehydrated in MeOH prior to *in situ* hybridization. For histological analysis, ovaries were embedded in JB-4 Plus plastic resin (Polysciences) and 5 micron sections were cut using a microtome. Sectioned ovaries were stained for 20 minutes with hematoxylin (Sigma-Aldrich), washed in distilled water, stained for 20 minutes with eosin Y (Polysciences), washed in distilled water, and cleared with 50% EtOH. Stained sections were coated with Permount (Fisher) and then coverslipped.

Whole mount *in situ* hybridization was performed as previously described [69]. Following staining, oocytes were embedded in JB-4 Plus Plastic resin and sectioned as described above. The *vasa* DIG labeled probe was generated using pTY27 [70] and the *dazl* probe was made using pCRII z*dazl* (gift from Kunio Inoue).

### Positional mapping of *mgn*

Genomic DNA was pooled from 18 mutant and 24 sibling (wild-type and heterozygous) females. Using these pools, *mgn* was mapped to chromosome 19 using SSLP markers that cover the genome as described [71]. The SSLP markers z31313 and z24515, which flank the *mgn* mutation, were used to genotype individual fish. SNP and SSLP markers were generated for further fine mapping. Fine mapping was performed using the SNP marker SNP3 (5′-CGT AGG CGT TGC ATA ACT GA-3′ and 5′-GCA AGC AAT CAT ACG CAC AT-3′) and either z53070 or CAmarker1 (5′-TTG AAG GGT CAC GTT TGA CA-3′ and 5′-CAA GGG TGA AGG GTG AAG AG-3′), which are approximately 7.7 kb apart. 976 meioses were analyzed, and 16 recombinants at SNP3 and 11 recombinants at z53070/CAmarker1 were used to narrow the interval to a 2.7 cM (2.02 Mb based on Ensembl Zv7/danRer5) interval.

### Region Specific Extraction (RSE)

For preparation of genomic DNA, fish were euthanized and then flash frozen in liquid nitrogen. 100 mg of tissue from each fish was homogenized and genomic DNA was isolated using the Qiagen Genomic-tip 100/G protocol (Qiagen).

Genomic DNA was enriched from two *mgn* mutant females by region specific extraction (RSE) [22], [72] for two overlapping regions: 1) A 319.8 kb interval on chromosome 19 (32,557,347–32,877,103; Zv7/danRer5) where the molecular lesion associated with the *mgn* phenotype was predicted to be based on meiotic mapping; and 2) A 613.9 kb interval on chromosome 19 (32,326,938–32,942,969; Zv7/danRer5) that extended the 319.8 kb region on both sides of *macf1* to examine flanking genes and identify SNPs. Genomic DNA from a wild-type (+/+) AB fish was also enriched for the 613.9 kb interval to identify SNPs to narrow the interval. RSE was carried out by Generation Biotech at CHOP/UPenn on a Qiagen BioRobot EZ1. For this capture technology, oligonucleotide primers are hybridized to targeted areas of the genome by exploiting sequence elements that are unique to the region of interest. The bound oligos are extended with biotinylated nucleotides to label the targeted DNA segments. Streptavidin coated magnetic microparticles are then added to the reaction mix to isolate the targeted DNA along with flanking regions [22], [72], [73].

The 30 microliter RSE reaction mix consisted of a pre-mixed set of targeting oligos (41 primers for the 319.8 kb region, 77 primers for the 613.9 kb region; oligo design and sequences are below) combined with 600 ng of genomic DNA. The genomic DNA was denatured and an automated capture performed, followed by washing and elution in pre-loaded reagent cartridges.

After RSE, the enriched DNA from each sample was removed from the microparticles by heating the solution at 80°C for 15 minutes to disrupt the biotin-streptavidin complex [22]. The microparticles were magnetically collected and the eluate, which contained the enriched material, was retained. The samples were then tested by quantitative PCR (QuantiTect) at three and six loci in the 319.8 kb and 613.9 kb target regions, respectively, and at the *beta-actin 2* locus on chromosome 3 as off-target. These quantitative assays showed enrichment of the target region with low amounts of off-target material in each sample. Specifically, for each 25 microliter reaction, 8 microliters of sample was combined with 1X Qiagen Quantitect Probe PCR master mix (Cat # 204345), 0.4 micromoles each of forward and reverse primers (Integrated DNA Technologies, IDT, Iowa) and 0.2 micromoles probe (IDT). Six 1:3 serially diluted zebrafish genomic DNA standards were run in duplicate for each locus as well as a single negative control. Forty cycles of 95°C for 15 seconds, 60°C for 1 min were run after the initial denaturation at 95°C for 15 minutes. Fluorescence was collected at 60°C. The primer sequences are:

#### Target qPCR assays primers and probes

5′-CCCTCGTTTTACTCCAGTCTAG-3′

5′-CTCCCTAAAATTCCTGTCCC-3′

5′-/56-FAM/TCCCATGTCACCCTGTCCCAAA/3BHQ_1/-3′

5′-GTTGCCCCAGTGTTGAAA-3′

5′-CACCCGTTTTCATTCGCA-3′

5′-/56-FAM/TTTTGCCGTGCTTGACTGATGGAG/3BHQ_1/-3′

5′-TAGGGAGACATTGAAGGGAAGGGT-3′

5′-TGCTGATCTTTGACCTCTCACACG-3′

5′-/56-FAM/GCCATGTAGCATCTTTCGCACAGTTCCA/3BHQ_1/-3′

The three additional target regions assayed for the 613.9 kb enrichment:

5′- GTGTTTCCTCTGAGTCATG -3′

5′- AGTGTTAGACCTCTGTGC -3′

5′-/56-FAM/ CCATGGGCCTTAAATGTGGTTTATCC/3IABLFQ/ -3′

5′- CCTGCATATCTTCATGGC -3′

5′- TCATCCTTACCTTCCACG -3′

5′-/56-FAM/ CCGTAAGCCTTTACATATCTCCAGCC/3IABLFQ/ -3′

5′- ACTGTGCGTTCTCAAGGA -3′

5′- GCCAGTTTCGCAGTCATT -3′

5′-/56-FAM/ TGGCATCCTAACCAGACATGTGACTC/3IABLFQ/ -3′

#### Off-target qPCR assay

5′-TTCGAGACCTTCAACACC-3′

5′-ACCATCACCAGAGTCCAT-3′.

5′-/56-FAM/TGTGCTGTCCCTGTATGCCTCC/3BHQ_1/-3′

The enriched samples were then amplified with Illustra GenomiPhi V2 DNA amplification kit (GE Healthcare Cat. # 25-6600-30) according to manufacturer's protocol. Residual primers and dNTPs were deactivated with ExoSAP-IT (USB Cat #78200) according to manufacturer's protocol. For each sample, approximately 2 micrograms of enriched, amplified material was used as input for preparation of the sequencing library. The library was prepared for sequencing using the Illumina Genomic DNA Sample Prep Kit (catalog # FC-102-1001). Sequencing was performed using an Illumina Genome Analyzer II. Consensus sequences were built using Maq software and analyzed using Sequencher (Gene Codes Corporation).

### Oligo design

A custom software program was used to identify the oligos for the enrichment. It utilizes and integrates several free, open source or publicly available software solutions to automatically generate appropriate oligo sets at user-defined intervals, spanning genomic regions of interest. In this experiment, 41 capture oligos were used to target the 319.8 kb region (Table 1) and 77 oligos for the 613.9 kb region (Table 2).

**Table 1:** Oligo Sequences for 319.8 kb capture.

Start position	Sequence
32557347	GAGTGCGTTTGGTTTCTGACGAAGA
32565483	ACACAGCATGGAGCCAGATAGACAA
32573772	TCCGCTGGTTTCCTCTTGTATGTCT
32581764	TGCAGAAACTCCTCCAACTCACCAT
32589761	CGTCACTCATGTTTGTGGATGTGCT
32598047	GGCTGATAACTCTGCACTGAGACCT
32605292	GGGAAAATGATCCACAGGTCCAGGT
32613439	AGACACAGCGCCTTTATTACCTCCA
32622385	TACATCACACACTGGCATCTGGGT
32629186	CGGGAAAAGAGGATTTACCCAGGGA
32637220	AGCCCGTTCTTCTAGGTTCTTGAGG
32645745	GAGGACATCTGAAATGCTTGGAGCC
32654005	TGAGTTTGTAAGTGAGCTGTGCTGC
32661561	AGCACTCTTCTTGCAGTGGCTCTAT
32669516	TTACCTGATGTGCGACAAGTGATGG
32677260	GGTTCTCCAGGCATCTAATCTCGCT
32685963	GTTCTGGTGTTAAGGATCTGCGAGC
32693461	GCAGCAAGTCCTCTAATCTGCCAAG
32702671	ATGCAACGGAGGTGAGGTTTGTGA
32709282	ATCGTACATGAGCTGCAATGCCTTC
32718576	GTCGCTGAGATGCAACGTGTACTT
32726077	TGATTTGCTCAACCCCTTGCTCAAC
32733197	GATGAACCACGACTGTCATGCCCTT
32741331	TTTGACTTTGCCAGGAACACCAGC
32749953	ATCCCAACGATGTGCACCTGAAAAG
32757988	AACTGCTTGTCACATGCTCCACATC
32766047	CAAACTGACAAGGTTGCCCTGATGT
32774043	ACTAGCCCAGACCTGTGTGATCCTA
32781606	TTTGTCAAATCCAGCCACCTGACAC
32789661	GGACCTGGCGGGCATTAAACAATAG
32797206	TCGTCAGATAGGACTGAAAACGCCA
32805201	CCCTCCAGACACTCGAAGTTGTCATA
32813828	TTTGCTTTGGGGAGGATTGCATCAG
32821258	TGACTCCCTCCTTCGTATTGACAGC
32829736	GCAGGACAGGATGAGTGATGTGAAC
32837643	CTGTGACCCGCTGCTAATGTTTCAA
32845311	GAGCCACCCCTGTTTAAGCATTCAT
32853241	CACACAGTTGCCAGATTGCTTCGTA
32861615	ATTATGCCTGCATGTGAAGGGAAGC
32869968	TGTGATGGTTGGCGTGACTTCATTC
32877081	TGACATTTACGGACAGCATCGG

**Table 2:** Sequences for additional oligos used for 613.9 kb capture.

Start position	Sequence
32327580	GAAGTTGTGCGGTGCATTCTGGATA
32335279	AGAGTAAGACACTGACCCGAAACCG
32344111	GGTTGTTTATCCAGAGTGTCCGCA G
32352587	TCTGCGTCGAGTGTATGTGGTAGAG
32359757	GCAACGAATCTGTGTAAGGTGACGA
32368565	AGGCTTCCAATGTCAACAGATCCCT
32375887	CCAGGCACTACTTCACAGTTTCAGG
32382569	CACACGCTGATTTGATGCTTCTGGA
32392099	GGCTGGTAGTGTCTTTGATGGAAGC
32399141	TGGAGTTTGACCTCACATACCTGCT
32407627	AAGGAAGTGACCAAAACAGCCCATC
32415234	TATCTTCACACCACATCCAGCCCAT
32423604	ATACCTGGCTGTATGCTCCCTCATG
32430251	CGGTCATTCACAAGCGGAAGAGATG
32438734	AACGAACTTTCCACCAGCAACCATC
32448247	ACATGCAACCCAAATAAGACAGGAAGT
32454980	TCTGCTGACTTCCATCTAACTGTGC
32462988	GTGACATAAAGCCCATAACCCTGCC
32470115	CAGTGATCGTCTCATGGGTGTCTCA
32479464	TGTAGCCCATGCCCTGAATGAAGTA
32490870	TCCCATACCTGCAAATCCTTGTCCA
32495691	GATGTGAATAATCGGTCCTGCCCAC
32503310	ACGTGACTGTGCGTCTATAATGGGA
32511589	TGAGACATTATCCACCGACACCGTC
32518509	TGTTGCAGTGAGGAGAATGTGTGGA
32529058	GTCTTGACGCCGAGGTATTGTCTCT
32535546	AGGCTTTAGGCACTTCATCTGGTCT
32544250	ACCGCTGCTGAGTTTCGCATCTATA
32890292	ATTGCACTGACCTCACTCTGTAGGG
32893147	CTGCTTCTCGAATACCCGTCCAGTT
32901203	GTCACACACTGCACTTTCACACACT
32909819	ACCTCCTCAACACTGACTTATGCGT
32915780	CCACTGAAGATGCGTCATGCTACTG
32925279	GCAGATGCTTACTACACACACTTAC
32933665	CAGACTCACAATCATCAGCCTTTGC
32941519	TGCAAGCACAATCATAAGCCAAGTT

The oligos were designed to target unique zebrafish sequence spanning the region of interest at approximately 8 kb intervals. Through the functionalities embedded in the software program, the UCSC web browser (http://genome.ucsc.edu) was first accessed to retrieve repeat- and SNP-masked DNA sequences for the target region in order to exclude these positions as capture points. The program is designed in such a way that the user enters a large genomic region (currently up to 1 Mb), which is then parsed into smaller regions in which the oligos are designed. After generating the oligos, the user is able to redesign small regions in the event that the oligo selection was poor or missing for that smaller region. The preliminary oligo set was then checked via Blat against a local Blat server to ensure that the selected sequences only match to one region. Currently the local Blat server supports human, mouse and zebrafish genomes; more genomes can be added.

In a final step, the software program was used to test for cross-reactivity to ensure minimal homo- and hetero-dimerization and hairpin formation. This step ensures that all selected oligos will work well together in a multiplexed format. The GC content for the final oligo set was between 48–52% and the Tm range was 57–61°C.

### Calculation of enrichment

Enrichment values were calculated using the following formula:

(Reads that map to the region examined/total number of reads)/(size of interval examined/genome size). Enrichment was calculated based on an estimated genome size of 1.6 Gb. For the first sequence capture, enrichment of *mgn* mutant DNA = (280884/5861492)/(315606/1.6×10^9^) = 243. For the second sequence capture, enrichment of wild-type DNA = (430216/15480724)/(616031/1.6×10^9^) = 72. For the second capture, enrichment of *mgn* mutant DNA = (259817/15639239)/(616031/1.6×10^9^) = 43.

### Immunohistochemistry and fluorescent labeling

Ovaries were dissected from euthanized females and treated with 3 mg/ml collagenase for 10 minutes in Medium 199 (Invitrogen) to improve post-fixation morphology (the majority of follicle cells were still present). For acetylated tubulin staining, ovaries were then fixed in 4% paraformaldehyde for 2 hours at room temperature, washed, dehydrated in MeOH and then stained essentially as described [74]. Anti-acetylated tubulin antibody (Sigma) was used at a dilution of 1:350. For rhodamine phalloidin staining, dissected ovaries were fixed overnight at 4°C in Actin Stabilizing Buffer and stained as described [75]. Dissected ovaries were fixed for DiOC_6_ staining in 4% paraformaldehyde overnight at 4°C. Following washes in PBS, ovaries were stained in 5 micrograms/ml DiOC_6_ (Calbiochem) for 2 hours at room temperature or overnight at 4°C. Confocal microscopy was performed using a Zeiss LSM 510 Laser Scanning Inverted Microscope. Images of acetylated microtubule staining were median filtered using ImageJ to decrease background.

Oocyte and Balbiani body sizes were calculated using measurements made with ImageJ software. Because fixed oocytes are not uniformly round, Balbiani body and oocyte diameters were determined by measuring the areas of each from single confocal sections and then calculating the diameters of circles based on the areas. For each oocyte, the optical section with the largest oocyte area was used to measure oocyte size, and the section with the largest Balbiani body area was used to measure Balbiani body size. For calculations of nucleus position, the centroids of the oocyte and the nucleus were determined from single confocal sections using ImageJ. For these studies, Excel was used to calculate statistical significance, which was determined by performing a two-sample unequal variance t-test where alpha = 0.05.

### Electron microscopy

Ovaries from *mgn* mutant or heterozygous siblings were dissected and fixed in 2.5% glutaraldehyde+ 2% paraformaldehyde in 0.1 M Sodium Cacodylate overnight at 4°C. Fixed samples were rinsed in 0.1 M Sodium Cacodylate buffer, post-fixed with 2% osmium tetroxide, dehydrated in graded ethanol, and embedded in Epon. 70 nm thin sections were stained with uranyl acetate and bismuth sub-nitrite [76], [77]. Stained sections were examined with a JEOL JEM 1010 electron microscope and imaged with a Hamamatsu CCD camera and AMT 12-HR software. Reagents and supplies were purchased from Electron Microscopy Sciences, Fort Washington, PA.
